# Trametinib boosts palbociclib’s efficacy in breast cancer via autophagy inhibition

**DOI:** 10.32604/or.2024.046139

**Published:** 2024-06-20

**Authors:** ANGUO WU, JIAO YAN, TING SU, CHI FENG, XIN LONG, YIRU PAN, RUPEI YE, TIAN XIA, HANAN LONG, JIANMING WU, XIULI XIAO

**Affiliations:** 1 Sichuan Key Medical Laboratory of New Drug Discovery and Druggability Evaluation, Luzhou Key Laboratory of Activity Screening and Druggability Evaluation for Chinese Materia Medica, Key Laboratory of Medical Electrophysiology of Ministry of Education, School of Pharmacy, Southwest Medical University, Luzhou, 646000, China; 2 The Department of Pathology, The Affiliated Hospital of Southwest Medical University, Luzhou, 646000, China; 3 The Department of Pathology, Mianyang Central Hospital, School of Medicine, University of Electronic Science and Technology of China, Mianyang, 621000, China; 4 The Department of Pathology, Yongchuan Hospital of Chongqing Medical University, Chongqing, 402160, China; 5 School of Basic Medical Sciences, Southwest Medical University, Luzhou, 646000, China; 6 School of Clinical Medical Sciences, Southwest Medical University, Luzhou, 646000, China

**Keywords:** Palbociclib, Trametinib, Protective autophagy, RAF/MEK/ERK, MCF7, MDA-MB-468

## Abstract

Breast cancer, a predominant global health issue, requires ongoing exploration of new therapeutic strategies. Palbociclib (PAL), a well-known cyclin-dependent kinase (CDK) inhibitor, plays a critical role in breast cancer treatment. While its efficacy is recognized, the interplay between PAL and cellular autophagy, particularly in the context of the RAF/MEK/ERK signaling pathway, remains insufficiently explored. This study investigates PAL’s inhibitory effects on breast cancer using both *in vitro* (MCF7 and MDA-MB-468 cells) and *in vivo* (tumor-bearing nude mice) models. Aimed at elucidating the impact of PAL on autophagic processes and exploring the potential of combining it with trametinib (TRA), an MEK inhibitor, our research seeks to address the challenge of PAL-induced drug resistance. Our findings reveal that PAL significantly decreases the viability of MCF7 and MDA-MB-468 cells and reduces tumor size in mice while showing minimal cytotoxicity in MCF10A cells. However, PAL also induces protective autophagy, potentially leading to drug resistance via the RAF/MEK/ERK pathway activation. Introducing TRA effectively neutralized this autophagy, enhancing PAL’s anti-tumor efficacy. A combination of PAL and TRA synergistically reduced cell viability and proliferation, and *in vivo* studies showed notable tumor size reduction. In conclusion, the PAL and TRA combination emerges as a promising strategy for overcoming PAL-induced resistance, offering a new horizon in breast cancer treatment.

## Introduction

Breast cancer stands as a leading cause of cancer-related morbidity and mortality among women globally, posing a significant challenge to public health [1]. Despite strides in understanding its molecular basis and developing diverse treatments, breast cancer remains a complex challenge for oncologists and researchers. This complexity arises mainly from the disease’s heterogeneity, the evolution of drug resistance, and the adverse effects of current treatments [2–4]. Our study aims to addresses these during challenges by exploring innovative combination therapies, with focusing on the role of autophagy in drug resistance.

A shift toward personalized medicine and targeted therapies has significantly evolved in the treatment paradigm for breast cancer [5]. Our research probes the synergistic effects of combining palbociclib (PAL), a cyclin-dependent kinase 4/6 (CDK4/6) inhibitor, with trametinib (TRA), an ERK inhibitor. This novel strategy aims to counteract resistance mechanisms induced by PAL in breast cancer treatment. PAL has emerged as a significant component in targeted therapies, especially effective in hormone receptor-positive (HR^+^) breast cancers. Nonetheless, resistance to PAL, whether intrinsic or acquired over time, significantly impedes its long-term efficacy [6,7].

Recent studies highlighting autophagy’s role—a cellular process for degrading and recycling cellular components—underscore its contribution to resistance development against treatments like PAL [8]. Autophagy enables cancer cells to survive under stress, potentially counteract negating the antiproliferative effects of PAL and leading to therapeutic resistance [8]. Our study investigates the combined influence of PAL and TRA on modulating autophagy and the RAF/MEK/ERK signaling pathway in breast cancer cells. By inhibiting ERK signaling, TRA emerges as a potential strategy to overcome autophagy-mediated resistance linked with PAL.

Our findings indicate that the PAL and TRA combination not only reduces PAL-induced autophagy but also synergistically boosts the anti-cancer effects of PAL, both *in vitro* and *in vivo*. This suggests that targeting both cell cycle and autophagy pathways may be an effective strategy in breast cancer therapy, potentially overcoming drug resistance and improving patient outcomes. This study contributes to the growing body of research on combination therapies in breast cancer, offering new insights into overcoming drug resistance and paving the way for more effective and personalized treatment approaches.

## Materials and Methods

### Chemicals, antibodies, and plasmids

TRA, rapamycin (RAPA), bafilomycin A1 (Baf), and PAL were obtained from Topscience Co. (Shanghai, China) and dissolved in dimethyl sulfoxide (DMSO) to create stock solutions at appropriate concentrations for *in vitro* assays. These solutions were freshly prepared immediately before use. The crystal violet staining solution was purchased from Saint-Bio (Shanghai, China). Antibodies used for immunoblotting and immunohistochemistry were meticulously selected for their established specificity and reliability. The primary antibodies included LC3B (3868S), p-RAF (2962S), RAF (14814S), p-MEK (9154S), MEK (4694S), p-ERK (4370S), and ERK (4695S) from Cell Signaling Technologies (Beverly, MA, USA); β-actin from Sata Cruz Biotechnology (Dallas, Texas, USA); GAPDH from Proteintech (Rosemont, IL, USA). Secondary horseradish peroxidase (HRP)-conjugated anti-rabbit and anti-mouse antibodies were purchased from Medical & Biological Laboratories Co., Ltd. (Nagoya, Japan). The pEGFP-LC3 plasmid (#11546) was acquired from Addgene (Cambridge, MA, USA).

### Cell culture

The MCF7 cell line, a human breast adenocarcinoma cell line, was sourced from the American Type Culture Collection (Manassas, VA, USA). MDA-MB-468 cells and MCF10A cells were obtained from the Key Laboratory of Epigenetics and Oncology of Southwest Medical University. MCF7 and MDA-MB-648 cells were cultured in Dulbecco’s Minimum Essential Medium (DMEM), while MCF10A cells were cultured as previously described. Stable RFP-GFP-LC3 U87 cells provided by Dr. Xiaoming-Zhu (Macau University of Science and Technology) were cultured in α-MEM. All media were supplemented with 10% fetal bovine serum (FBS) and 1% penicillin-streptomycin from Gibco (Waltham, MA, USA). Cells were maintained at 37°C in a humidified atmosphere with 5% CO_2_.

### MTT assay

Cellular viability was evaluated using the widely employed 3-(4,5-dimethylthiazol-2-yl)-2,5-diphenyltetrazolium bromide (MTT, Sigma, MO, USA) assay, a colorimetric technique reflecting metabolic activity and cell proliferation [9]. Briefly, MCF7 cells, MDA-MB-468 cells or MCF10A cells were seeded into 96-well plates to ensure optimal growth and adherence. After cell attachment, various concentrations of TRA, PAL, or their combination were introduced, and cells were incubated for specified time intervals to allow drug exposure. Subsequently, the growth media were aspirated. Cells were then gently washed with PBS, and fresh growth media supplemented with MTT reagent solution were added to each well. After a 4-h incubation, the MTT-containing media were gently removed, and DMSO was added to dissolve the formazan crystals formed within the cells. The absorbance of the resulting formazan solution was measured spectrophotometrically at 570 nm using a microplate reader (BioTek, Winooski, USA). Absorbance values were normalized to control samples, and cell viability percentage was calculated using the formula: (Absorbance of treated sample/Absorbance of control sample) × 100.

### Colony formation assay

To assess the long-term effects of test drugs on MCF7 and MDA-MB-468 cell survival and clonogenic potential, a colony formation assay was conducted [10]. MCF7 cells and MDA-MB-468 cells were seeded into 6-well plates at appropriate densities and treated with test drugs for 6 days. After the incubation, the culture media were aspirated, and cells were gently rinsed with PBS. Fresh growth media without any drug treatments were added to each well, and cells were allowed to grow for an additional 6 days. Once suitable colony sizes were achieved, the media were carefully removed, and colonies were fixed and stained with the crystal violet solution. High-resolution images of the entire well were captured using a camera.

### Quantification of GFP-LC3B puncta formation

The formation of GFP-LC3B puncta, indicative of autophagosome accumulation, was quantified in MCF7 and stable RFP-GFP-LC3 U87 cells [11]. MCF7 cells, transfected with the pEGFP-LC3 plasmid, along with stable RFP-GFP-LC3 U87 cells, were cultured on cover slides in 6-well plates. Twenty-four hours later, the cells were treated with test drugs at specified concentrations. Post-treatment, the cells were fixed, mounted, and imaged using a Nikon ECLIPSE 80i fluorescence microscope (NIKON, Tokyo, Japan). The average number of GFP-LC3B puncta per cell was analyzed and quantified using ImageJ software (NIH, Bethesda, MD, USA), employing a predetermined threshold for puncta size and fluorescence intensity.

### Western blot

Post-treatment, cells or tumor tissue were harvested and lysed using RIPA buffer, supplemented with protease and phosphatase inhibitors. Protein concentrations were determined, and equal amounts of protein were subjected to SDS-PAGE electrophoresis. The separated proteins were then transferred onto PVDF membranes and blocked with 5% BSA in TBST solution. The membranes were incubated with primary antibodies targeting specific proteins of interest at recommended dilutions. This was followed by incubation with secondary HRP-conjugated antibodies. Protein bands were visualized using ECL reagent (4A Biotech, Beijing, China) and detected by the ChemiDoc MP Imaging System (Bio-Rad, Hercules, CA, USA). Densitometric analysis of the protein bands was performed using ImageJ software, with quantification normalized to GAPDH or β-actin.

### Subcutaneous tumor model establishment and drug administration

The mouse tumor model was established by subcutaneously implanting MCF7 cells into nude mice. Cells, prepared at a concentration of 2.5 × 10^7^ cells/mL, were transplanted within 30 min of digestion. Mice were acclimated for a week prior to the experiment. Tumors were initiated by injecting 0.2 mL of cell suspension into the right axillary region after disinfection. Once the tumor volume reached 100 mm^3^, tumor-bearing mice were allocated for drug administration. To assess PAL’s tumor-suppressive effect *in vivo*, tumor-bearing nude mice were randomly divided into four groups (*n* = 6 per group): control (normal saline), PAL (50 mg/kg, 100 mg/kg, and 150 mg/kg). For assessing the combined tumor-suppressive effect of PAL and TRA, tumor-bearing mice were divided into four groups (*n* = 5 per group): control (normal saline), PAL (100 mg/kg), TRA (1 mg/kg), and a combination of PAL (100 mg/kg) and TRA (1 mg/kg). Intragastric administration was conducted daily for 14 days, with body weight and tumor volume monitored every 3 days. Post-treatment, mice were euthanized, and tumor tissues were collected for further experiments. All animal experiments were approved by the Animal Experimental Ethics Committee of Southwest Medical University (Approval No. SWMU202005-17).

### Immunohistochemistry analysis

Formalin-fixed, paraffin-embedded breast cancer tissue specimens were prepared for immunohistochemical analysis [12]. Sections, 4–5 μm thick, were cut using a microtome and mounted onto positively charged adhesive-coated glass slides. Heat-induced epitope retrieval was performed using a citrate buffer solution (pH 6.0), tailored to the specific antibodies used. The slides were then cooled to room temperature. Non-specific binding sites were blocked using a protein-blocking agent. The slides were incubated with primary antibodies specific to target proteins for 24 h at 4°C. After washing off excess primary antibodies, the sections were incubated with species-specific secondary antibodies conjugated to HRP. The unbound secondary antibodies were washed away, and signal amplification was achieved through the application of enzyme substrates like 3,3′-diaminobenzidine (DAB). Concurrently, nuclear counterstaining with hematoxylin facilitated morphological assessment and localization of immunoreactivity. Immunostained slides were examined under a light microscope (Nikon, Tokyo, Japan), and the captured images were analyzed quantitatively or semi-quantitatively using ImageJ analysis software.

### Hematoxylin-Eosin (H*&*E) staining

Formalin-fixed, paraffin-embedded breast cancer tissue sections (4–5 μm thick) were deparaffinized in xylene and rehydrated using decreasing concentrations of ethanol [10]. Hematoxylin staining was conducted to reveal cellular nuclei, involving immersion in Hematoxylin solution to selectively bind the dye to the nuclei. After Hematoxylin staining, the sections were rinsed, and differentiation in tap water was performed to remove excess stain, enhancing nuclear contrast. “Bluing” of the nuclei was achieved by brief immersion in an alkaline solution. After rinsing, Eosin counterstain was applied to color the cytoplasm and extracellular structures. The sections were then dehydrated in ascending ethanol concentrations, followed by clearing in xylene or substitute. Finally, they were mounted using a permanent mounting medium and covered with a coverslip. Once dried, representative images of the mounted sections were captured and analyzed using a light microscope.

### Statistical analysis

The experimental data were subjected to meticulous statistical examination using GraphPad Prism software (version 9.0). Descriptive statistics, including the calculation of mean and standard deviation (SD), were employed to succinctly summarize the data distribution. The significance among the groups was determined through a one-way analysis of variance (ANOVA), followed by Tukey’s *post hoc* analysis for multiple pairwise comparisons. A significance threshold of *p* < 0.05 was established to determine statistical significance.

## Results

### PAL inhibits breast cancer in vitro and in vivo

**Figure 1:**
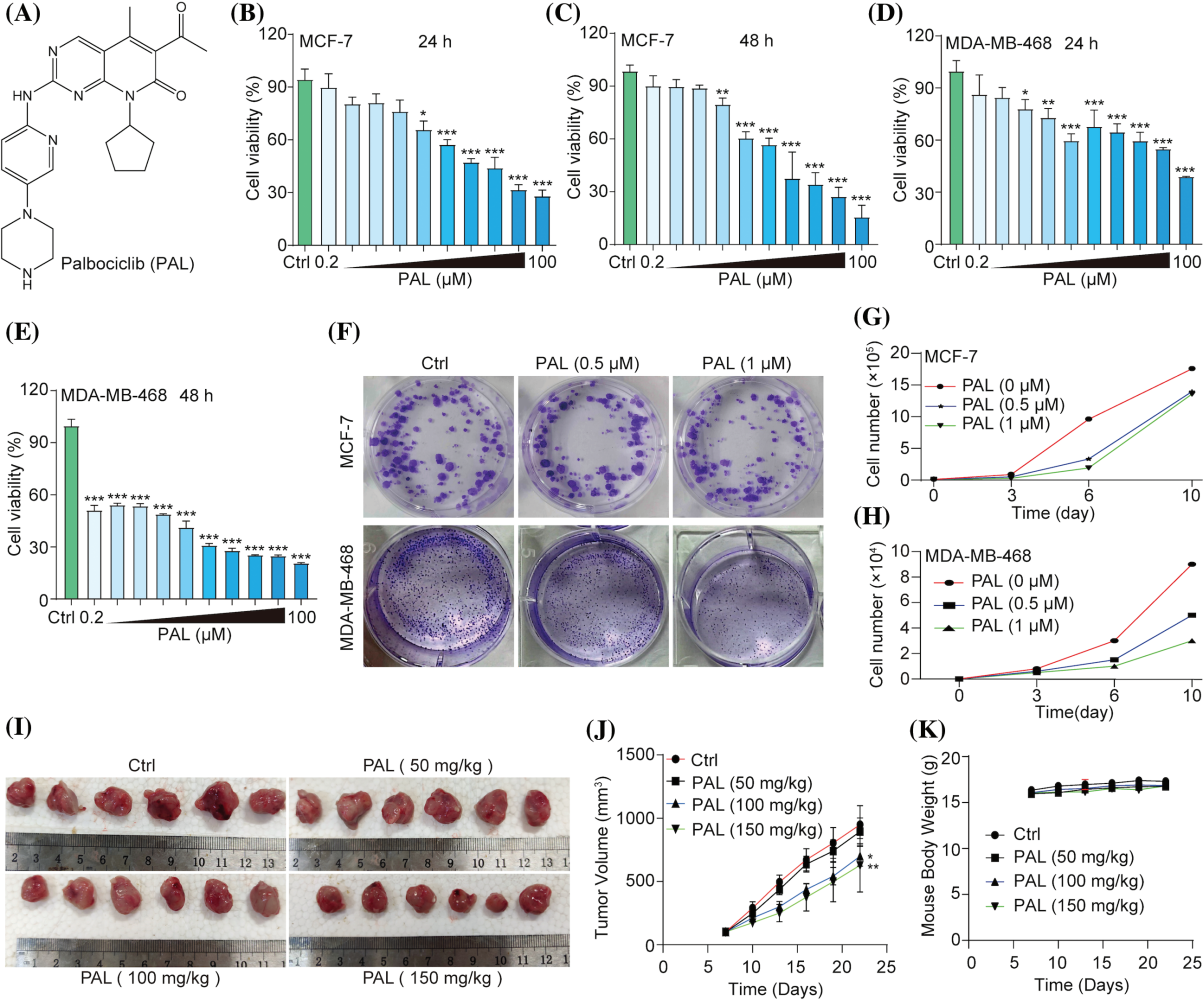
PAL inhibits breast cancer *in vitro* and *in vivo*. (A) The chemical structure of PAL. (B, C) MTT assay results showing the cell viability of MCF7 cells treated with PAL at indicated concentrations for 24 and 48 h. (D, E) MTT assay results for the cell viability of MDA-MB-468 cells treated with PAL at indicated concentrations for 24 and 48 h. (F) Representative crystal violet staining images depicting the colony formation of MCF7 and MDA-MB-468 cells treated with PAL at indicated concentrations. (G, H) The curve lines indicate the cell number of MCF7 and MDA-MB-468 cells treated with PAL at indicated concentrations over 3, 6, and 10 days. (I) Images showing the appearance of MCF7 tumor extracted from nude mice treated with either vehicle or PAL. (J) A curve line graph depicting the tumor volumes in vehicle- and PAL-treated mice. (K) A curve line graph showing the body weight changes in vehicle- and PAL-treated mice. Bar. S.D., *p < 0.05, **p < 0.01, ***p < 0.001.

In an initial evaluation of PAL’s inhibitory capacity, (Fig. 1A), its effects on breast cancer were examined both *in vitro* and *in vivo*. The cell viability of MCF7 cells and MDA-MB-468 cells was observed to decrease in both a dose- and time-dependently manner when treated with PAL. The IC_50_ values were 12.73 μM at 24 h and 8.72 μM at 48 h for MCF7 cells (Figs. 1B, 1C), and 1.21 μM at 24 h and 0.61 μM at 48 h for MDA-MB-468 cells (Figs. 1D, 1E). Additionally, the cytotoxicity of PAL on MCF10A cells, representing normal human breast cells, was examined and found to be minimal (Suppl. Fig. S1). Using crystal violet staining, a significant decrease in colony formation was noted in MCF7 and MDA-MB-468 cells upon exposure to PAL (Fig. 1F). This finding was further confirmed by direct cell counting, which highlighted PAL’s strong cytotoxic effects on both MCF7 and MDA-MB-468 cells. Specifically, reductions of 65.3% and 80.2% in MCF7 cells (Fig. 1G), and 44.4% and 66.7% in MDA-MB-468 cells were observed at PAL concentrations of 0.5 μM and 1 μM, respectively (Fig. 1H). *In vivo* experiments conducted on tumor-bearing nude mice corroborated these findings, demonstrating noticeable reductions in tumor size after PAL administration compared to untreated control mice (Fig. 1I). Quantitative data further substantiated this observation, indicating a significant decrease in tumor volume in the PAL-treated group (Fig. 1J). Importantly, the consistent body weights of the mice throughout the study suggested that the administered doses of PAL did not induce systemic toxicity (Fig. 1K). In summary, the data illustrate that PAL exerts potent inhibitory effects on breast cancer both *in vitro* and *in vivo*.

### PAL triggers protective autophagy in breast cancer

**Figure 2:**
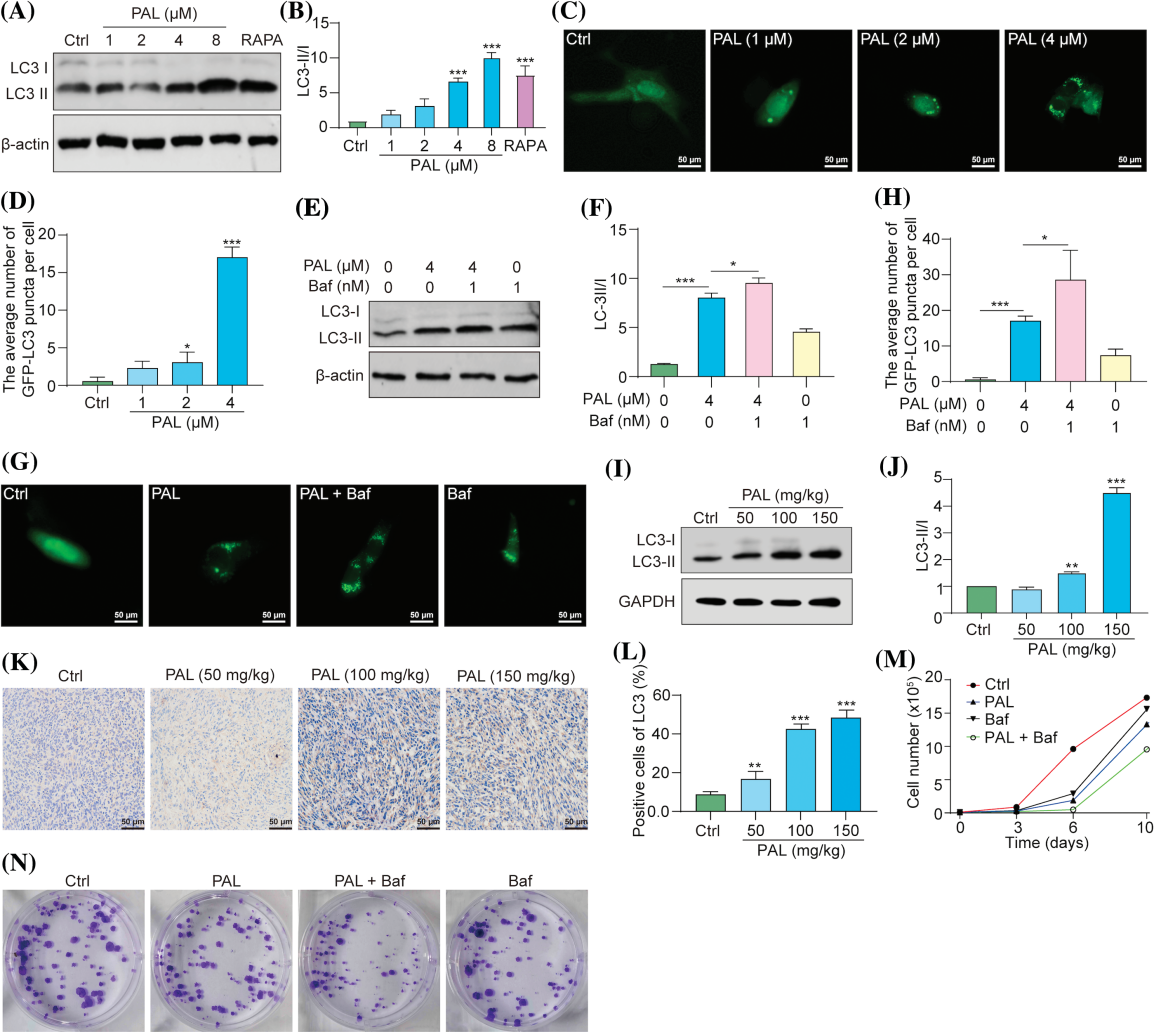
PAL triggers protective autophagy in breast cancer. (A) Western blotting analysis of LC3 from the protein lysate of MCF7 cells treated with RAPA and PAL at indicated concentrations. (B) A bar chart showing the ratio of LC3-II to LC3-I. (C) Representative images of pEGFP-LC3 transfected MCF7 cells treated with PAL at indicated concentrations. Magnification: 40×. Scale bar: 50 μm. (D) A bar chart showing the average number of GFP-LC3 puncta per cell. (E) Western blotting analysis of LC3 from the protein lysate of PAL-treated MCF7 cells, with or without Baf. (F) A bar chart indicating the ratio of LC3-II to LC3-I. (G) Representative images of EGFP-LC3 transfected MCF7 cells treated with PAL in the presence or absence of Baf. Magnification: 40×. Scale bar: 50 μm. (H) A bar chart showing the average number of GFP-LC3 puncta per cell. (I) Western blotting analysis of LC3 from the protein lysate of tumor tissue extracted from nude mice treated with either vehicle or PAL. (J) A bar chart indicating the ratio of LC3-II to LC3-I. (K) Representative immunohistochemical analysis images of LC3 from sections of tumor tissue. Magnification: 40×. Scale bar: 50 μm. (L) A bar chart showing the percentage of positive cells for LC3 expression. (M) A curve line graph indicating the number of PAL-treated MCF7 cells in the presence or absence of Baf. (N) Representative crystal violet staining images depicting the colony formation of PAL-treated MCF7 cells in the presence or absence of Baf. Bar. S.D., *p < 0.05, **p < 0.01, ***p < 0.001. Full unedited gel/blots are provided in Suppl. Fig. S2, where the protein molecular weight markers were labeled.

Drug resistance poses a significant challenge in cancer treatment, including targeted therapies like PAL for breast cancer [13]. Moreover, autophagy, a cellular process responsible for degrading and recycling cellular components, can either suppress or promote tumor growth, depending on the context [14]. In our study, we evaluated the autophagic activity induced by PAL in MCF7 cells and tumor samples. Using Western blotting assays, we investigated the autophagic activity initiated by PAL in MCF7 cells. Notably, there was a clear shift from LC3-I to LC3-II in PAL-treated MCF7 cells (Figs. 2A, 2B). PAL also significantly increased the average number of GFP-LC3 puncta in MCF7 cells transfected with the pEGFP-LC3 plasmid (Figs. 2C, 2D). To assess the autophagic flux of PAL-treated MCF7 cells, we employed Baf, a well-known autophagy inhibitor. When treated simultaneously with PAL and Baf, a pronounced increase in the conversion of LC3-I to LC3-II and a higher count of GFP-LC3 puncta were observed, compared to MCF7 cells treated with PAL alone (Figs. 2E–2H). Moreover, tumor samples from PAL-treated mice were analyzed using Western blotting and immunohistochemical staining. These analyses revealed an increased LC3-I to LC3-II conversion and LC3B protein expression, consistent with *in vitro* findings (Figs. 2I–2L). To explore the potential role of PAL-induced autophagy in relation to its inhibitory effectiveness on breast cancer, Baf was used alongside PAL. Subsequent cell counting and colony formation tests demonstrated a significant reduction in MCF7 cell count and colony formation (Figs. 2M, 2N), highlighting the synergistic efficacy of Baf and PAL. In conclusion, our findings suggest that PAL induces protective autophagy in breast cancer.

### PAL activates the RAF/MEK/ERK signaling pathway in breast cancer

**Figure 3:**
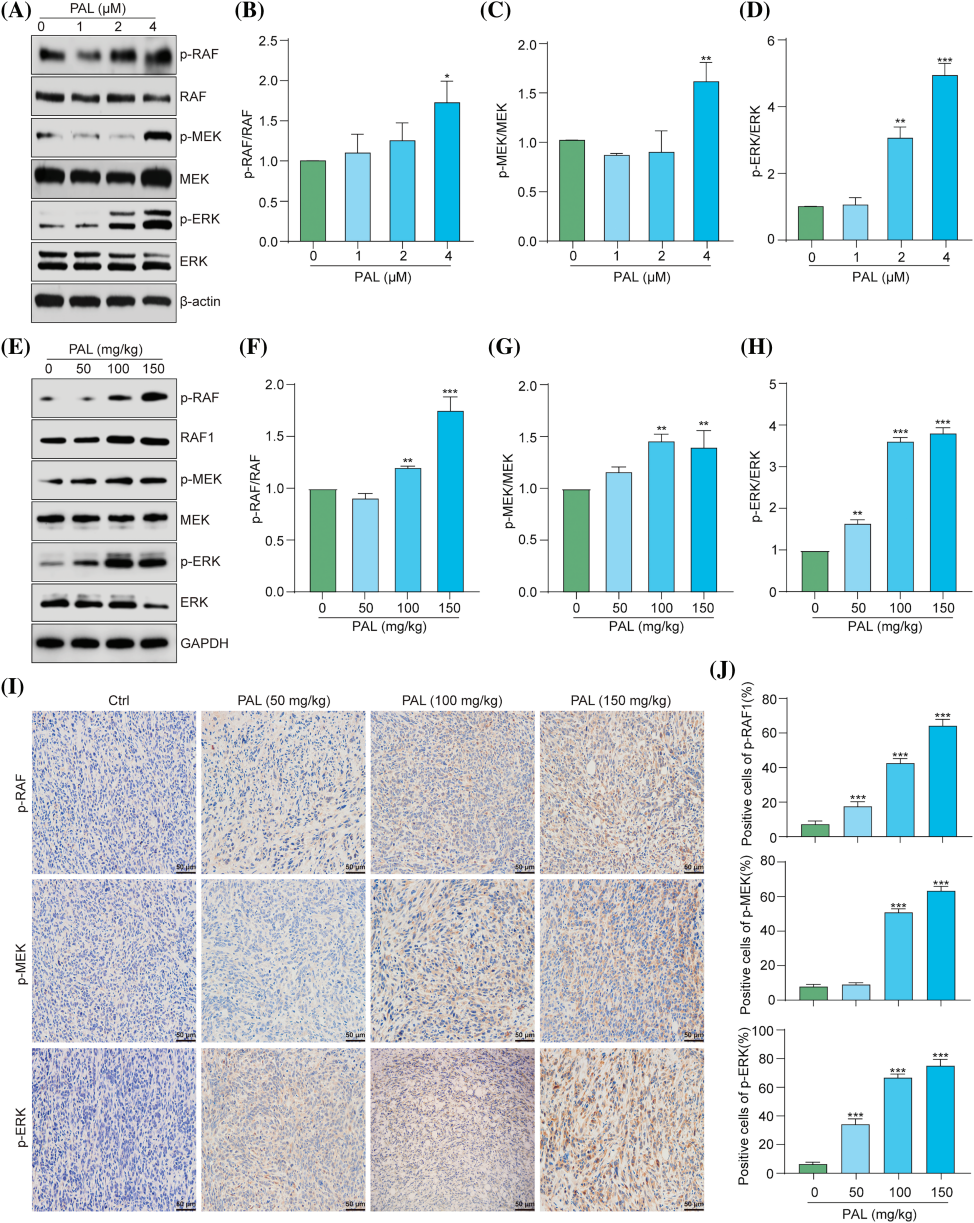
PAL activates the RAF/MEK/ERK signaling pathway in breast cancer. (A) Western blotting analysis of p-RAF, RAF, p-MEK, MEK, p-ERK, and ERK from the protein lysate of PAL-treated MCF7 cells. (B–D) Bar charts displaying the ratios of p-RAF/RAF, p-MEK/MEK, p-ERK/ERK in MCF7 cells. (E) Western blotting analysis of p-RAF, RAF, p-MEK, MEK, p-ERK, and ERK from the protein lysate of tumor tissue extracted from nude mice treated with either vehicle or PAL. (F–H) Bar charts showing the ratios of p-RAF/RAF, p-MEK/MEK, and p-ERK/ERK in tumor tissue. (I) Representative immunohistochemical analysis images of p-RAF, p-MEK, and p-ERK in sections of tumor tissue. Magnification: 40×. Scale bar: 50 μm. (J) Bar charts indicating the percentage of cells positive for p-RAF, p-MEK, and p-ERK expression. Bar. S.D., *p < 0.05, **p < 0.01, ***p < 0.001. Full unedited gel/blots are provided in Suppl. Fig. S3, where the protein molecular weight markers were labeled.

The RAF/MEK/ERK pathway is crucial in regulating cellular processes, including growth, division, and survival [15]. Inhibiting this pathway is a potential therapeutic strategy, particularly for cancers with mutations in genes like BRAF [16]. In cancer, the induction of autophagy through the activation of the RAF/MEK/ERK pathway may lead to drug resistance [17]. Recent evidence suggests that PAL upregulates the ERK signaling pathway in certain cancers, and inhibiting this pathway could enhance the efficacy of PAL treatments [18–20]. This indicates that the activation of the RAF/MEK/ERK pathway might contribute to PAL-related drug resistance in breast cancer. To assess the impact of PAL on the RAF/MEK/ERK pathway, we performed Western blotting analysis on MCF7 cells and tumor tissue samples from breast cancer xenografts. Our results revealed increased phosphorylation of RAF, MEK, and ERK proteins in both PAL-treated MCF7 cells and tumor tissues (Figs. 3A–3H). Additionally, we conducted immunohistochemical staining of tumor tissue sections, focusing on the phosphorylated (p-) forms of RAF, MEK, and ERK within the tumor microenvironment. This staining demonstrated a notable increase in the levels of p-RAF, p-MEK, and p-ERK in PAL-treated tumor tissues (Figs. 3I, 3J). These findings suggest a direct link between PAL treatment and the activation of the RAF/MEK/ERK signaling cascade in breast cancer.

### TRA inhibits PAL-induced autophagy in breast cancer

**Figure 4:**
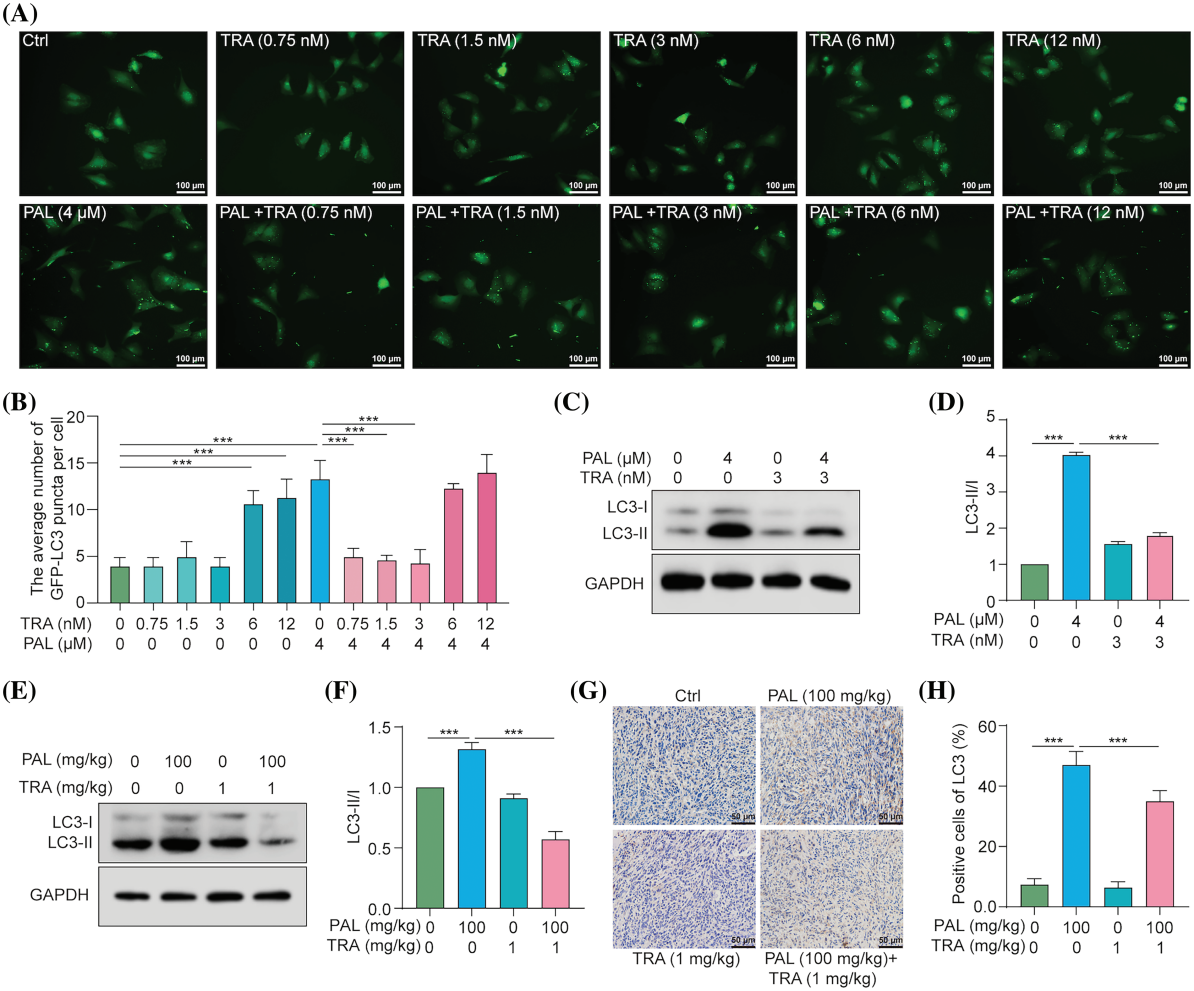
TRA inhibits PAL-induced autophagy in breast cancer. (A) Representative images depicting GFP-LC3 puncta in U87 cells treated with PAL in the presence or absence of TRA at indicated concentrations. Magnification: 20×. Scale bar: 100 μm. (B) A bar chart showing the average number of GFP-LC3 puncta per cell. (C) Western blotting analysis of LC3 from the protein lysate of PAL-treated MCF7 cells with or without TRA. (D) A bar chart indicating the ratio of LC3-II to LC3-I. (E) Western blotting analysis of LC3 from the protein lysate of tumor tissue extracted from nude mice treated with PAL, with or without TRA. (F) A bar chart showing the ratio of LC3-II to LC3-I in the tumor tissue. (G) Representative immunohistochemical analysis images of LC3 in tumor tissue from PAL-treated nude mice, with or without TRA. Magnification: 40×. Scale bar: 50 μm. (H) A bar chart indicating the percentage of cells positive for LC3 in the tumor tissue. Bar. S.D., ***p < 0.001. Full unedited gel/blots are provided in Suppl. Fig. S4, where the protein molecular weight markers were labeled.

To determine whether PAL induces autophagy in breast cancer by activating the RAF/MEK/ERK signaling pathway, we utilized TRA, a specific inhibitor of MEK, to counteract PAL’s effect on activating RAF/MEK/ERK. We used stable RFP-GFP-LC3 U87 cells and then treated them with PAL in the presence or absence of various concentrations of TRA. Fluorescence microscopy observations indicated that low concentrations of TRA (1.25–5 nM), but not high concentrations (≥10 nM), significantly decreased the number of GFP-LC3 puncta in U87 cells (Figs. 4A, 4B). This suggests that low concentrations of TRA inhibited PAL-induced autophagy. Additionally, Western blotting analysis revealed a marked decrease in the conversion of LC3-I to LC3-II when MCF7 cells were treated with both TRA and PAL. This reduction in LC3-I conversion supported the idea that low concentrations of TRA could suppress PAL-induced autophagy in MCF7 cells (Figs. 4C, 4D). In tumor tissue samples, Western blotting data showed a decrease in LC3-I conversion upon treatment with both TRA and PAL, as opposed to PAL alone (Figs. 4E, 4F). Furthermore, immunohistochemical staining showed reduced expression of LC3B in the tumor tissues of animals treated with the combination, confirming the inhibitory effect of TRA on PAL-induced autophagy *in vivo* (Figs. 4G, 4H).In summary, these findings indicate that PAL triggers autophagy by activating the RAF/MEK/ERK signaling pathway.

### TRA augments PAL sensitivity in breast cancer

**Figure 5:**
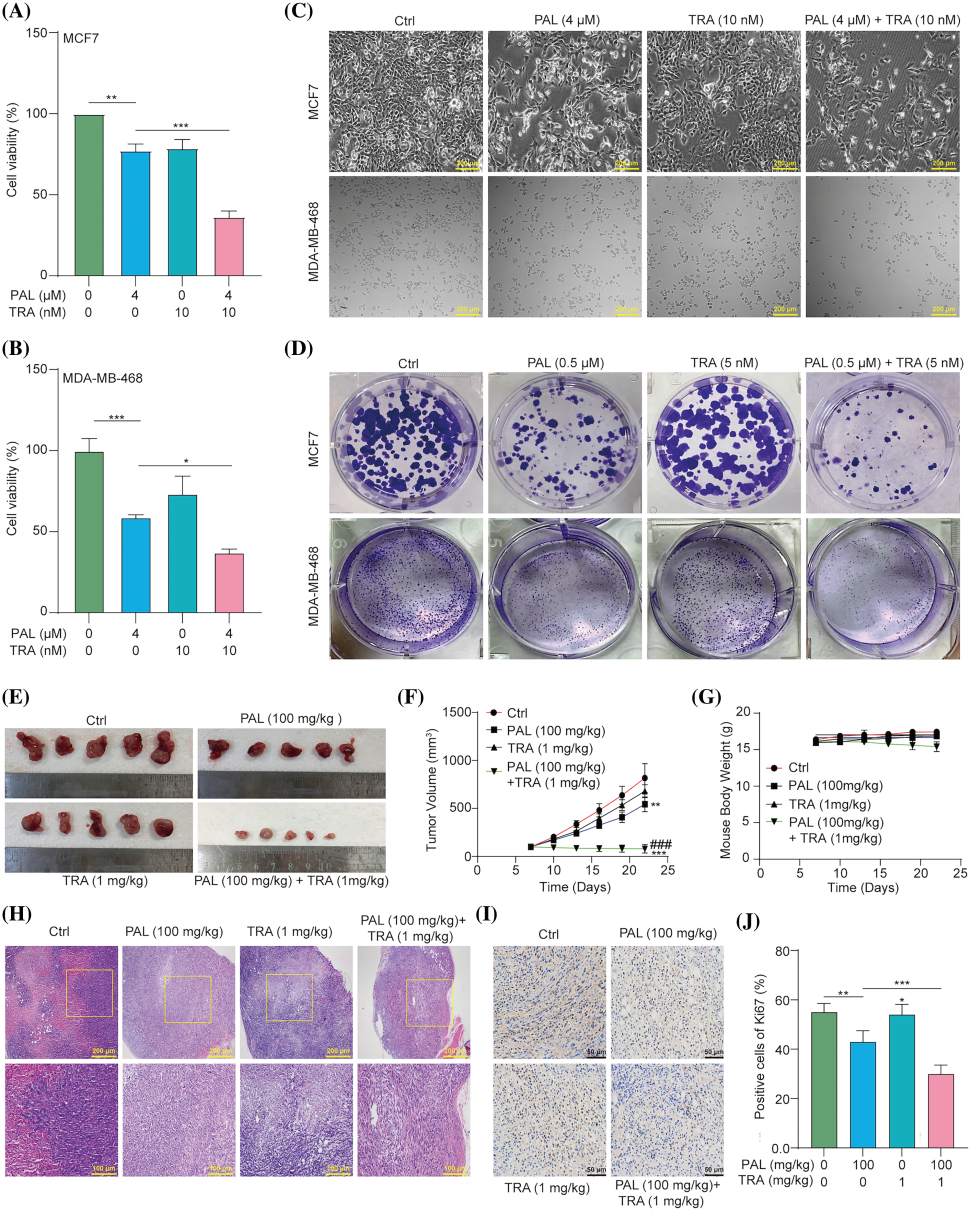
TRA augments PAL sensitivity in breast cancer. (A, B) Bar charts showing the cell viability of PAL-treated MCF7 and MDA-MB-468 cells with or without TRA at indicated concentrations for 24 h. (C) Representative images of cellular morphology images for PAL-treated MCF7 cells and MDA-MB-468 cells with or without TRA at indicated concentrations for 24 h. Magnification: 10×. Scale bar: 200 μm. (D) Representative crystal violet staining images displaying the colony formation of PAL-treated MCF7 cells and MDA-MB-468 cells with or without TRA at indicated concentrations. (E) Images showing the appearance of the MCF7 tumors extracted from nude mice treated with PAL, with or without TRA. (F) A curve line graph indicating the tumor volume of PAL-treated nude mice with or without TRA. (G) A curve line graph showing the body weight of PAL-treated nude mice with or without TRA. (H) Representative H&E staining images of tumor tissue from PAL-treated nude mice with or without TRA. Magnification: 10× (upper panel) and 20× (lower panel). Scale bar: 200 μm (upper panel) and 100 μm (lower panel). (I) Representative immunohistochemical analysis images of Ki67 in tumor tissue from PAL-treated nude mice with or without TRA. Magnification: 40×. Scale bar: 50 μm. (J) A bar chart indicating the percentage of cells positive for Ki67. Bar. S.D., *p < 0.05, **p < 0.01, ***p < 0.001, ^###^*p* < 0.001 *vs*. PAL (100 mg/kg).

To evaluate whether inhibiting the RAF/MEK/ERK signaling pathway enhances the efficacy of PAL in breast cancer treatment, we conducted an MTT assay to measure the cell viability of PAL-treated MCF7 and MDA-MB-468 cells in the presence or absence of TRA. The results showed that treating these cell lines with a combination of TRA and PAL resulted in a significant reduction in cell viability compared to treatment with either drug alone (Figs. 5A, 5B). The combined treatment demonstrated a synergistic effect, suggesting increased sensitivity of breast cancer cells to PAL when combined with TRA. Under the microscope, MCF7 and MDA-MB-468 cells treated with the drug combination exhibited noticeable morphological changes compared to controls, including increased cell shrinkage, membrane blebbing, and reduced cell density, indicative of enhanced cell death (Fig. 5C). Additionally, there was a significant decrease in the number of colonies in MCF7 and MDA-MB-468 cells treated with both TRA and PAL compared to cells treated with either drug alone (Fig. 5D), indicating augmented sensitivity and decreased proliferation. However, no significant changes were observed in the viability of MCF10A cells treated with either the drug combination or alone (Suppl. Fig. S5). In *in vivo* studies using nude mice with breast cancer tumors, the combination treatment significantly reduced tumor size and volume compared to treatment with either drug alone (Figs. 5E, 5F). During the treatment period, there was no significant difference in body weight among the different groups, suggesting that the drug combination did not cause systemic toxicity (Fig. 5G). H&E staining of tumor sections from animals treated with the combination revealed increased necrosis and reduced cellular density compared to treatment with either drug alone (Fig. 5H). Furthermore, tumor sections from combination-treated animals showed reduced Ki67 staining, indicating a lower rate of tumor cell proliferation (Figs. 5I, 5J). These findings support the hypothesis that combination therapy leads to increased tumor cell death and a more robust anti-proliferative effect on breast cancer, while maintaining minimal cytotoxicity *in vitro* and *in vivo*.

## Discussion

Breast cancer continues to be one of the most common malignancies globally, highlighting the need for contiguous research to understand its fundamental mechanisms and develop more effective treatment strategies [21]. In this study, we conducted a comprehensive investigation of PAL, a known CDK inhibitor, and its impact on the progression of breast cancer, examining its effects both *in vitro* and *in vivo*. Additionally, we explored the potential interactions and synergistic effects between PAL and TRA, particularly focusing on their combined influence on tumor cell viability and autophagy.

Our findings clearly demonstrate PAL’s strong inhibitory effect on breast cancer. The dose- and time-dependent reduction in MCF7 and MDA-MB-468 cell viability and the marked decrease in cell colony formation highlight PAL’s cytotoxicity. Additionally, the cytotoxicity of PAL on MCF10A cells was found to be minimal. The *in vivo* data further support this, with PAL-treated tumor-bearing mice showing significant tumor size reductions compared to untreated controls. Notably, consistent body weights across the study suggest that PAL dosages did not induce systemic toxicity. This is in line with previous research underscoring PAL’s effectiveness in inhibiting cancer cell proliferation by targeting CDKs crucial for cell cycle progression [22].

One of the notable findings from our research is the induction of protective autophagy by PAL in breast cancer cells. Autophagy, a process of cellular recycling, plays a dual role in cancer, acting either as a tumor suppressor or promoter, depending on cellular context [10,23]. Our observations, particularly the increase in LC3-I to LC3-II conversion and GFP-LC3 puncta, indicate that PAL might induce a form of autophagy that serves a protective function. This could potentially limit the full therapeutic efficacy of PAL. The observation that combining PAL with Baf, an inhibitor of autophagy, led to a substantial decrease in MCF7 and MDA-MB-468 cell count and colony formation further highlights the importance of this autophagic response. This finding is consistent with other studies that highlight the complex role of autophagy in cancer treatment and its implications for therapeutic outcomes [24].

The RAF/MEK/ERK signaling pathway is critical in regulating cellular processes, such as cell growth, division, and survival [25,26]. In cancer, the activation of this pathway can contribute to drug resistance, presenting a significant challenge for therapeutic efficacy [27]. Our study findings show an upregulation of the pathway following PAL treatment, suggesting it as a potential mechanism behind drug resistance. This upregulation is evidenced by increased phosphorylation levels of RAF, MEK, and ERK proteins observed in both our *in vitro* and *in vivo* experiments. This observed enhancement in the ERK pathway activation aligns with previous research that discusses the role of this signaling cascade in the progression of cancer and drug resistance, especially in the context of targeted therapies [28].

In an effort to counteract the autophagic response initiated by PAL, we focused on TRA, a specific inhibitor of the MEK enzyme which consequently affects the RAF/MEK/ERK signaling pathway [29]. The results from our study are promising: TRA effectively inhibited the autophagy induced by PAL, as demonstrated by the reduction in GFP-LC3 puncta and the conversion from LC3-I to LC3-II. This suggests that the RAF/MEK/ERK pathway plays a crucial role in the induction of autophagy by PAL in breast cancer cells. This conclusion is supported by existing literature that delves into the molecular mechanisms of CDK inhibitors in the treatment of breast cancer, shedding light on how these therapies can be further optimized [30]. Moreover, our investigation into the combined effects of TRA and PAL provided significant insights. The treatment combining both TRA and PAL led to a notable reduction in the viability of MCF7 and MDA-MB-468 cells, accompanied by morphological changes that are indicative of cell death. However, no significant cytotoxicity was observed in MCF10A cells treated with either the drug combination or alone. In *in vivo* experiments, this synergistic effect resulted in a substantial decrease in tumor size and volume, without any noticeable systemic toxicity. Histological examination of tumor sections from animals treated with the combination therapy revealed an increase in tumor cell death and a reduction in cell proliferation, highlighting the potential of this combined treatment approach in breast cancer therapy.

While our study presents promising findings, there are several limitations that need to be addressed. Firstly, our reliance on specific cell lines and a single animal model may not adequately represent the complex and diverse nature of breast cancer in humans. Therefore, it is crucial to conduct further studies using a broader range of models to validate the generalizability of our results. Another important aspect that remains unexplored is the long-term effects of PAL and TRA combination therapy, including the potential for developing resistance mechanisms. Understanding these dynamics is essential for the successful application of our findings in clinical practice. Additionally, while we have identified the RAF/MEK/ERK signaling pathway and autophagy as key factors in the effects of PAL and TRA, a more in-depth examination of these pathways and their interactions is necessary. This would provide a clearer understanding of the molecular mechanisms driving the observed outcomes. Finally, translating these preclinical findings into clinical practice involves significant challenges. Determining the optimal dosing strategies and assessing potential side effects in humans are critical steps that must be carefully considered in future clinical trials. Ensuring the safe and effective use of PAL and TRA in breast cancer therapy requires meticulous planning and execution of these trials, taking into account all these factors.

## Conclusion

In conclusion, our study provides substantial evidence of a synergistic effect when combining PAL and TRA in breast cancer treatment, particularly in terms of inhibiting autophagy and modulating the RAF/MEK/ERK signaling pathway. Our research shows that this combination therapy not only counteracts PAL-induced drug resistance but also significantly enhances anti-tumor efficacy in both *in vitro* and *in vivo* models. This points towards a promising direction for advancing breast cancer therapy, especially in addressing the challenges of drug resistance. While these findings are indeed promising, clinical trials are crucial to evaluate the safety and effectiveness of this combination in human subjects and to establish the optimal dosing and treatment protocols. Additionally, gaining a deeper insight into the molecular mechanisms behind the observed synergistic effects could pave the way for novel targeted therapies in breast cancer treatment. However, there are challenges ahead. One major challenge is the variability in patient responses, underscoring the importance of personalized treatment approaches in breast cancer therapy. Developing biomarkers to predict responses to this combination therapy could play a key role in tailoring treatments to individual patients. Furthermore, the long-term effects and potential side effects of using PAL and TRA in combination require thorough investigation to ensure patient safety and well-being.

## Supplementary Materials

Supplementary Figure 1. The cell viability of MCF10A cells treated with PAL at indicated concentrations for 24 and 48 hours.

Supplementary Figure 2 The original Western blotting images of Figure 2.

Supplementary Figure 3 The original Western blotting images of Figure 3.

Supplementary Figure 4 The original Western blotting images of Figure 4.

Supplementary Figure 5. The cell viability of PAL-treated MCF10A cells with or without TRA at indicated concentrations for 24 hours.

## Data Availability

All data are available from the corresponding author upon request.

## Supplementary Materials

The supplementary material is available online at https://doi.org/10.32604/or.2024.046139.
