# Supplementary figures and images for: Trametinib boosts palbociclib’s efficacy in breast cancer via autophagy inhibition
Source: Oncol Res. 2024 Jun 20;32(7):1197–207. doi: 10.32604/or.2024.046139 (PMC11209742; doi:10.32604/or.2024.046139)

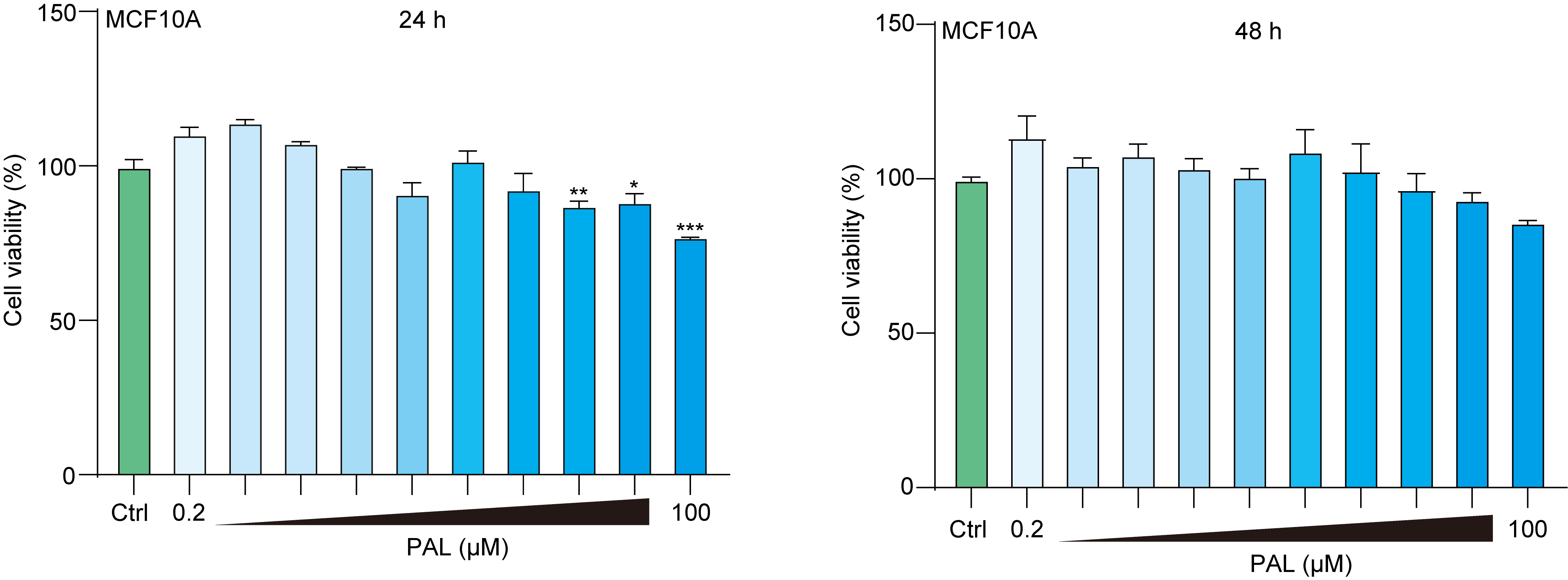

Supplement: Supplementary Figure 1. [file OncolRes-32-46139-s001.tif]

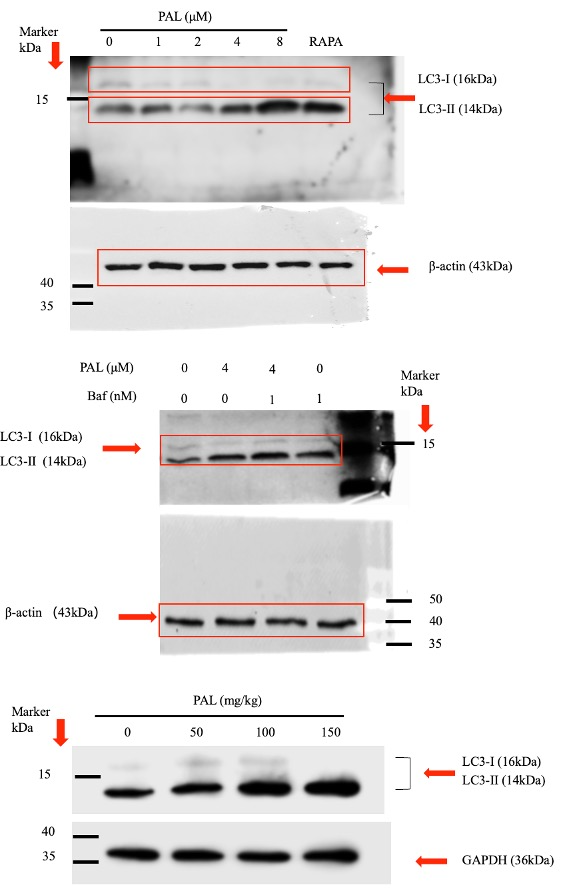

Supplement: Supplementary Figure 2 [file OncolRes-32-46139-s002.tiff]

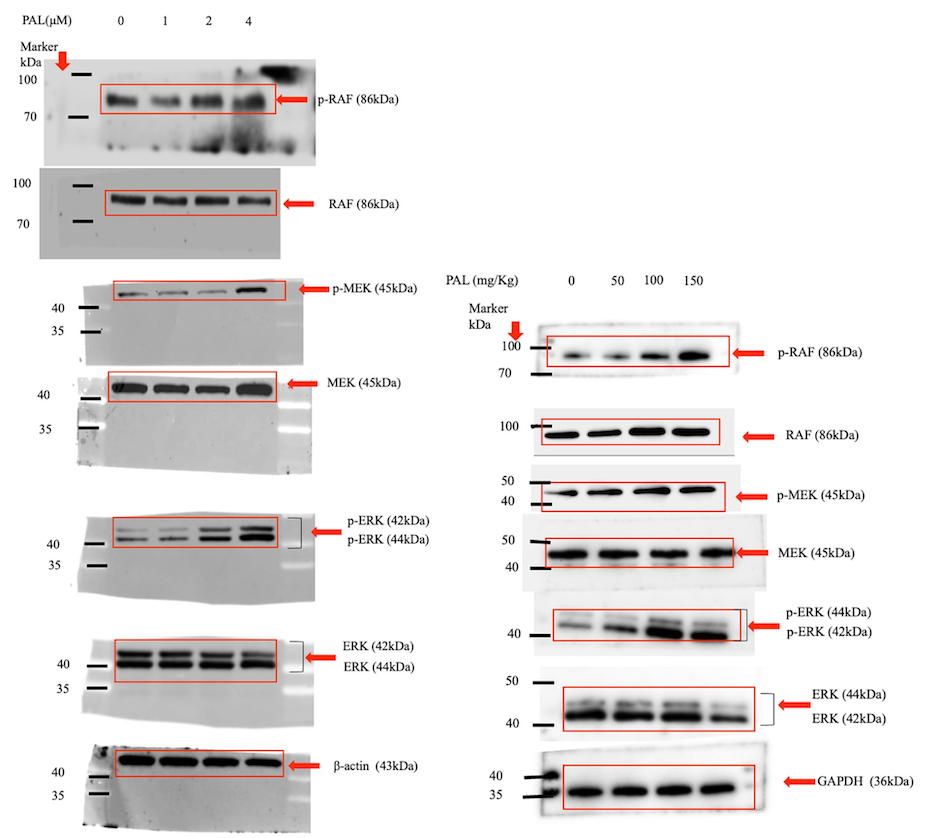

Supplement: Supplementary Figure 3 [file OncolRes-32-46139-s003.tiff]

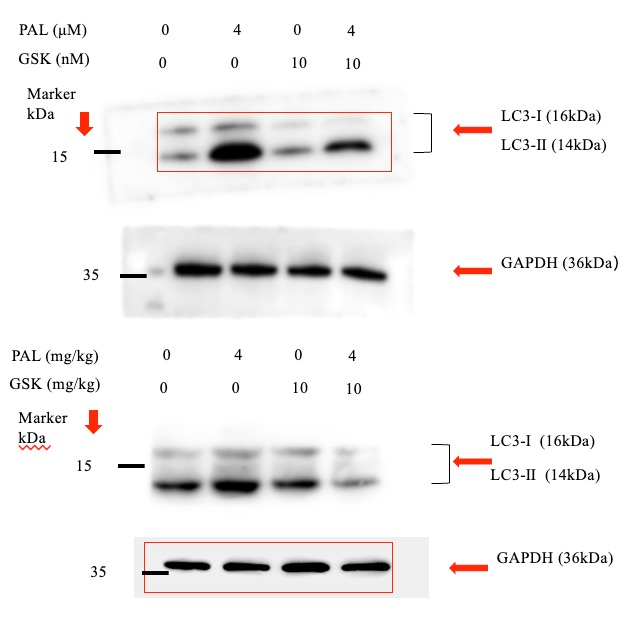

Supplement: Supplementary Figure 4 [file OncolRes-32-46139-s004.tiff]

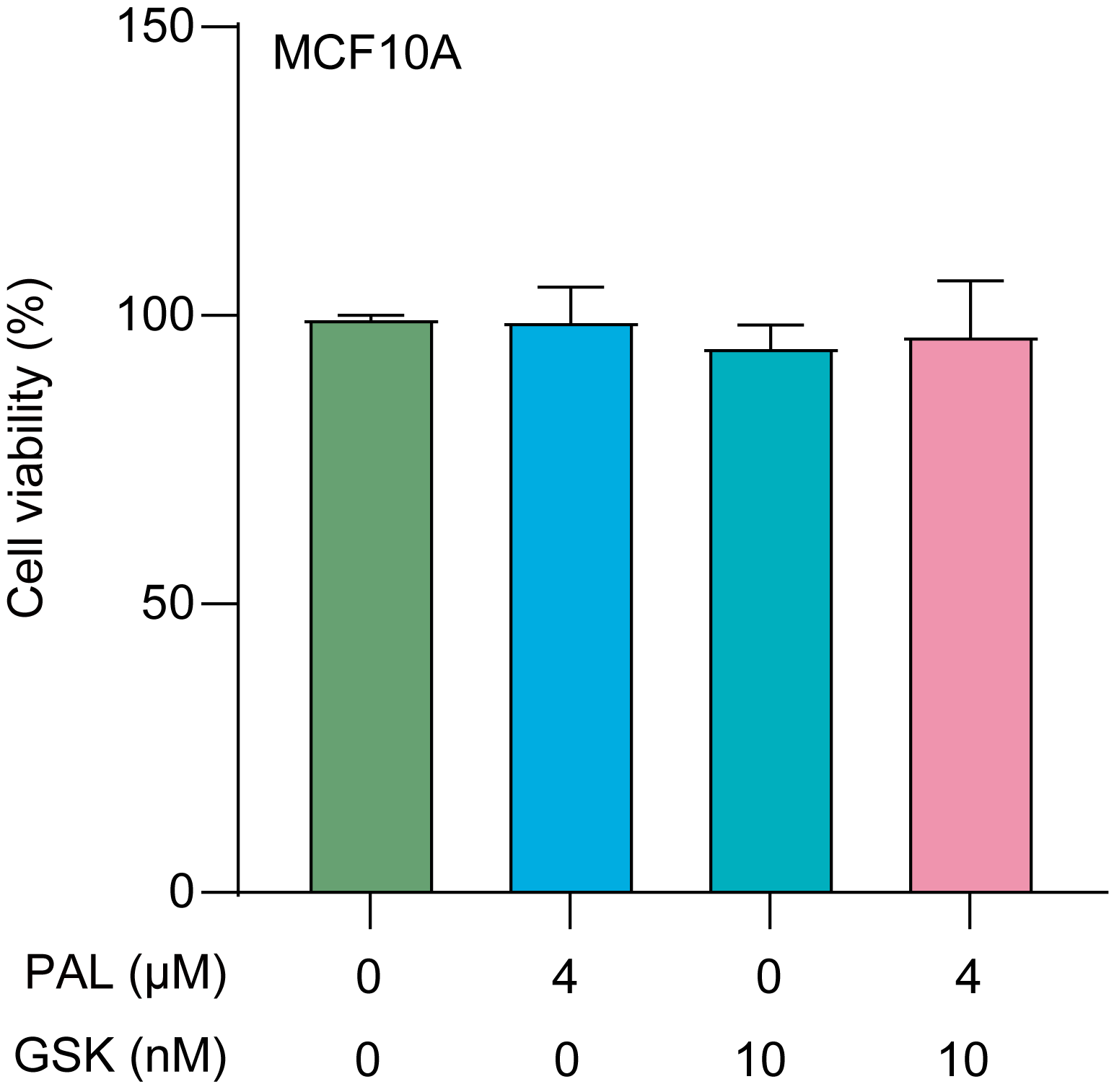

Supplement: Supplementary Figure 5. [file OncolRes-32-46139-s005.tif]
